# Natural bounds on herbivorous coral reef fishes

**DOI:** 10.1098/rspb.2016.1716

**Published:** 2016-11-30

**Authors:** Adel Heenan, Andrew S. Hoey, Gareth J. Williams, Ivor D. Williams

**Affiliations:** 1 Joint Institute for Marine and Atmospheric Research, University of Hawai‘i, Manoa, Honolulu, HI 96822, USA; 2 ARC Centre of Excellence for Coral Reef Studies, James Cook University, Townsville, Queensland 4811, Australia; 3 School of Ocean Sciences, Bangor University, Anglesey LL59 5AB, UK; 4 NOAA Pacific Islands Fisheries Science Center, Honolulu, HI 96818, USA

**Keywords:** fish biomass, functional group, herbivore, human drivers, natural drivers, Pacific Ocean

## Abstract

Humans are an increasingly dominant driver of Earth's biological communities, but differentiating human impacts from natural drivers of ecosystem state is crucial. Herbivorous fish play a key role in maintaining coral dominance on coral reefs, and are widely affected by human activities, principally fishing. We assess the relative importance of human and biophysical (habitat and oceanographic) drivers on the biomass of five herbivorous functional groups among 33 islands in the central and western Pacific Ocean. Human impacts were clear for some, but not all, herbivore groups. Biomass of browsers, large excavators, and of all herbivores combined declined rapidly with increasing human population density, whereas grazers, scrapers, and detritivores displayed no relationship. Sea-surface temperature had significant but opposing effects on the biomass of detritivores (positive) and browsers (negative). Similarly, the biomass of scrapers, grazers, and detritivores correlated with habitat structural complexity; however, relationships were group specific. Finally, the biomass of browsers and large excavators was related to island geomorphology, both peaking on low-lying islands and atolls. The substantial variability in herbivore populations explained by natural biophysical drivers highlights the need for locally appropriate management targets on coral reefs.

## 1. Introduction

Humans are increasingly a dominant global force influencing the structure and function of ecosystems through the removal of key species and functional groups, habitat modification, and the effects of pollution and climate change [1–3]. Coral reef ecosystems are especially vulnerable to such human-forcing [4], and whereas anthropogenic impacts are globally pervasive, they occur against a backdrop of high natural variability in reef systems caused by differences in the environment and biogeographic context. Oceanic productivity, water temperature, habitat area, reef geomorphology, and larval connectivity can have substantial impacts on coral reef fish assemblages [5–10]. For example, the natural fish carrying capacity of a coral reef has been shown to double along a gradient of increasing oceanic productivity [11]. Understanding the relative influence of human versus natural drivers is key to assessing the current status of these ecosystems.

Here, we focus on one component of coral reef systems, namely herbivorous fishes in the Pacific Ocean. Despite some uncertainty, particularly in the Indo-Pacific, about the relative importance of herbivory in mediating coral–algal dynamics [12–16], herbivorous fishes are widely recognized to play an important role in maintaining the competitive dominance of reef calcifiers (e.g. hard corals and crustose coralline algae), over other benthic components (e.g. fleshy macroalgae) [17–20]. For example, following climate-induced coral bleaching, fished reefs with reduced herbivore populations have a greater propensity to become dominated by macroalgae [21]. For that reason, some coral reef management strategies now focus specifically on protecting or restoring herbivorous fish populations [22,23]. There is a need, therefore, to better understand the role of the natural environment in determining distribution patterns of herbivorous fishes [8,24–26] independent of local human impacts on coral reefs. Indeed, the upper bounds of herbivore biomass will be determined by a reef's local biophysical setting, and once identified, will allow for realistic fisheries management strategies to address the widespread effect of fishing on this trophic group [7,8,11,27–30].

Herbivorous reef fish assemblages vary with local environmental factors. For instance, parrotfish tend to be more abundant and species rich on barrier reefs compared with atoll, and fringing or low coral cover reefs [31]. Intra-island variation in herbivore species composition and behaviour is also evident among different reef habitats. Typically, the abundance and feeding activity of grazing surgeonfishes and large parrotfishes is lower on nearshore coastal reefs compared with wave-exposed offshore reefs [32,33]. Conversely, browsing herbivores are often more abundant on wave-protected back reef habitats, when compared with the exposed fore-reef areas [32,34,35]. Furthermore, herbivore biomass and rates of herbivory tend to be the greatest on the reef crest, and both decrease across the reef flat and down the reef slope [35–38]. These patterns in herbivorous fishes are variously attributed to the availability and quality of food and shelter, in addition to the wave energy and sedimentation regimes experienced [34,38–40]. The implication of this localized among- and within-habitat variation is that the need for, and potential effectiveness of, fishery management interventions are highly dependent on natural bounds set by the location's biophysical setting [41].

Here, we make use of a consistent monitoring dataset from 33 islands and atolls across the central and western Pacific to better understand the relative role of anthropogenic impacts and biophysical drivers (habitat and physical environmental conditions) in structuring herbivore populations on coral reefs. These islands span large gradients of human population density (0–27 people per hectare of reef) [11,42] and biophysical condition [43], allowing us to separate the relative effect of those in driving variation in herbivore biomass.

## 2. Methods

### (a) Fish assemblage and reef habitat surveys

We used coral reef monitoring data collected between 2010 and 2015 across 33 Pacific islands and atolls (electronic supplementary material, table S1). The surveys were performed for the National Oceanic and Atmospheric Administration (NOAA) Pacific Reef Assessment and Monitoring Programme (Pacific RAMP), a long-term ecosystem monitoring effort focused on United States and United States-affiliated coral reefs [44]. Data from two underwater visual census techniques were used, the stationary point count (SPC) and the towed-diver (tow) survey method (Coral Reef Ecosystem Program; Pacific Islands Fisheries Science Center (2016). National Coral Reef Monitoring Program: stratified random surveys (StRS) of reef fish, including benthic estimate data of the U.S. Pacific Reefs since 2007. NOAA National Centers for Environmental Information. Unpublished Dataset. [15 August 2016], https://inport.nmfs.noaa.gov/inport/item/24447. Coral Reef Ecosystem Program; Pacific Islands Fisheries Science Center (2016). Towed-diver surveys of large-bodied fishes of the U.S. Pacific Reefs since 2000. NOAA National Centers for Environmental Information. Unpublished Dataset. [15 August 2016], https://inport.nmfs.noaa.gov/inport/item/5568). The SPC was used to estimate the biomass of herbivorous fishes, whereas the latter was used to estimate biomass of large (more than 50 cm in total length) piscivores. Piscivore biomass was used to investigate what effect, if any, piscivores may have in exerting top-down control on herbivore populations [45]. The tow estimates of piscivore biomass were used in preference to the SPC owing to the concern that small-scale surveys can overestimate the biomass of large roving predators, such as sharks and jacks [46].

A total of 3 309 SPC surveys were conducted by experienced surveyors. Survey site locations were selected per sampling unit (typically an island/atoll, occasionally, a cluster of small islands or for large islands, island subsection) by means of a randomized stratified design [47]. The target sampling domain of Pacific RAMP is hard bottom habitat in depths less than 30 m, and site allocation is stratified by reef zone (fore reef; back reef; lagoon) and depth (0–6 m; 6–18 m; 18–30 m). Only data from the fore-reef habitat were used to remove any biases associated with interhabitat differences on herbivorous fish assemblages; the fore reef is the most comparable reef habitat present across all islands. At each survey site, a pair of divers conducted simultaneous adjacent counts in which they first compiled lists of all fish species present within their survey area (7.5 m radius cylinder) during a 5 min interval. After the timed interval, divers proceeded to count and size all fishes from the species list within their survey area. Divers then visually estimated benthic cover and reef complexity, the mean vertical substrate height from the reef plane in the survey cylinder.

A total of 861 tow surveys were analysed. Surveys were haphazardly located on reef areas at a depth of 10–20 m, with the broad goal of spreading sites as widely as possible around each island; circumnavigating the island where practical. A pair of divers (one fish, one benthic surveyor) were towed behind a small boat travelling approximately 2 km for each 50 min survey. During each tow, the fish diver recorded the number and size of all species more than 50 cm in total length within a belt-transect extending 5 m on either side and 10 m in front of the diver, from the seafloor to the surface. Full details on the tow survey method are available in [46].

### (b) Data processing

The weight per individual fish was calculated using length-to-weight relationships from FishBase and other sources [48,49]. To date, much of the evidence of human impacts on herbivore populations relative to regional biophysical variation considers these herbivorous fishes as a single trophic guild or broad taxonomic groups [8], although, see [24]. Collectively, these studies point to differences in the expected richness and biomass of herbivorous fishes, either *in toto* or of specific families, based on habitat, island type, and biogeographic region [7,8]. There is, however, increasing evidence that different herbivore functional groups perform complimentary roles in reef processes [50], have different dietary and habitat requirements [8,51,52], and are likely to respond differently to local biophysical settings. Therefore, we classified herbivorous fishes functionally (*sensu* [53]) and incorporated new dietary data specific to the study area. Five groups resulted: ‘browsers’, ‘grazers’, ‘detritivores’, ‘large excavators/bioeroders', and ‘scrapers/small excavators’ (electronic supplementary material, S2).

For the SPC surveys, site-level herbivorous fish biomass (g m^−2^), hard coral cover, and reef complexity were calculated by averaging the two diver replicates conducted at each site location. Data were inspected for site-level outliers, site-level observations of any of the fish metrics that were more than 97.5% of the interquartile range, were capped at that 97.5% value (electronic supplementary material, S3.1). Island-scale averages of the site-level metrics were calculated, first by averaging values within each depth stratum per island, and then weighting the mean estimates by the total area of each stratum per island [54,55]. Island-level tow estimates of piscivore biomass were calculated as equally weighted means of each tow per island across years. Species richness per functional group was estimated by generating species accumulation curves for each island, using the rarefaction method in the R package *vegan* [56].

### (c) Quantifying human and biophysical predictors

We used the published estimates of the following human and biophysical covariates, derived at the island level: human population density, chlorophyll a (mg m^−3^) as a proxy for phytoplankton biomass and oceanic productivity, total area of reef habitat, sea-surface temperature (SST °C), wave energy (kW m^−1^), and island type (electronic supplementary material, table S3.2). Island types were based on geomorphology, and classed as either high (e.g. basalt island) or low lying (e.g. carbonate island or atoll). Islands were also grouped by region (Hawaii, Central Polynesia, Gilbert, Ellis, and Marshall Islands, and Tropical Northwest Pacific [57]).

To determine anthropogenic impacts on herbivorous fishes, we used human population density (the number of people resident per island (from the 2010 US census) divided by the area of fore-reef habitat per island from Geographic Information System (GIS) habitat layers maintained by Pacific RAMP (electronic supplementary material, S.3.3). For the remote-sensing data, we used the lower climatological mean of SST from the Pathfinder v.5.0 dataset, and the climatological mean of chlorophyll a derived from the moderate resolution imaging spectroradiometer. The wave energy metric used was the climatological mean from NOAA's Wave Watch III wave model. Details on the methods used to generate island-specific biophysical metrics are described in full in [43].

### (d) Modelling

We fitted the generalized additive mixed-effects models (GAMMs) of island-level herbivore biomass (electronic supplementary material, S3.1) in R (www.r-project.org), using the *gamm4* package [58]. All models included region as a random effect to account for autocorrelation among islands within regions [59]. Wake is the only replicate in the Marshall, Gilbert, and Ellis Islands region, therefore, we report summary fish metrics for Wake (biomass and richness) but excluded it from the statistical modelling (total number of island replicates = 33). For total fish biomass and functional group biomass separately, we fitted GAMMs for all possible combinations of the predictor variables using the *UGamm* wrapper function, in combination with the *dredge* function in the *MuMIn* package [60].

We calculated Akaike's information criterion, corrected for small sample size (AICc) and the AICc-based relative importance weights (*w_i_*) to assess the conditional probability of each model. We report the model-average estimates for each predictor term based on the top-ranked models for each fish metric, top-ranked models being those with more than 0.05 Akaike weight. To test for influential data points and to check for model stability, we performed a jack-knife sensitivity test, calculating the percentage of times sequentially deleting single response variable data points produced the same top-ranking model structure (*sensu* [61]).

Finally, to visualize the effect of predictor terms on the herbivorous fish responses, we used the coefficients from the top-ranked models for each response variable separately to generate a predicted dataset. We set all other predictor terms to their median value then generated smoother terms for the predictor of interest and plotted these against the untransformed, unscaled fish metrics [11].

## 3. Results

**Figure 1.**
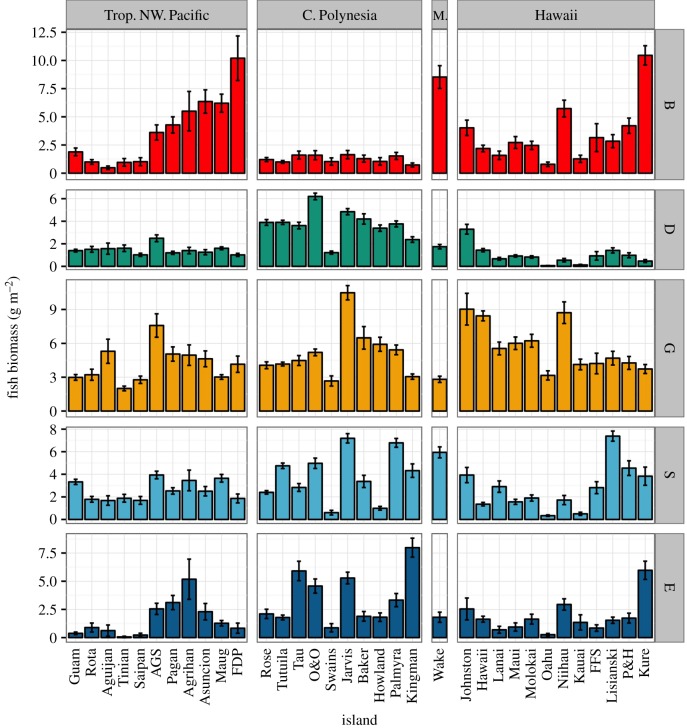
Herbivorous fish biomass by functional group per region. B, browsers (red), D, detritivores (green), G, grazers (yellow), S, scrapers and small excavators (blue), E, large excavators and bioeroders (dark blue). Trop. NW. Pacific, Tropical Northwest Pacific; C. Polynesia, Central Polynesia; M., Marshall Islands. AGS, Alamagan, Guguan, and Sarigan; FDP, Farallon de Pajaros; O&O, Ofu and Olosega; FFS, French Frigate Shoals; P&H , Pearl and Hermes. Islands within region are ordered by latitude. (Online version in colour.)

Across the western central Pacific, a large degree of variability exists in the biomass and composition of herbivorous fish assemblages, including the species richness within functional groups. Generally, there is greater biomass and richness of detritivores in Central Polynesia, and a greater biomass of browsers in the unpopulated northerly latitudes (figure 1 and electronic supplementary material, S4.1). Functional group biomass and richness was positively related in large excavators/bioeroders, scrapers/small excavators, and detritivores (electronic supplementary material, figure S4.2 and table S4.2).

**Figure 2.**
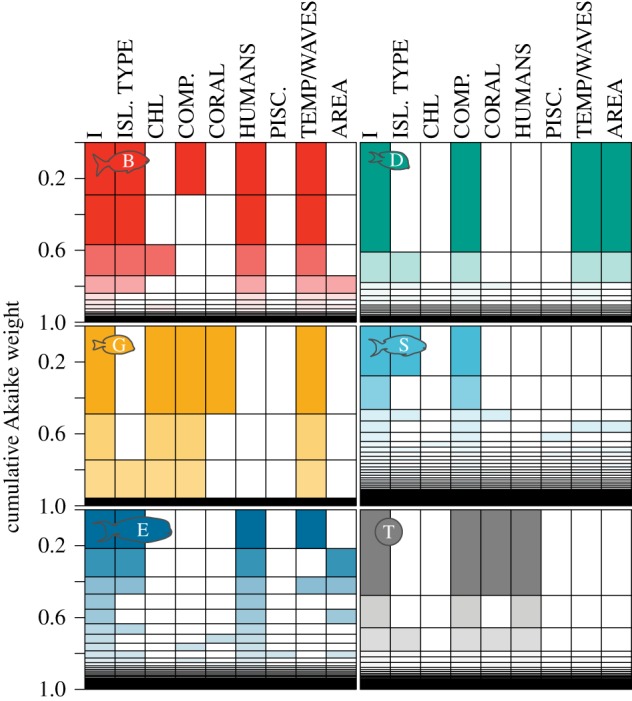
Model performance of generalized additive mixed-effects models (GAMMs). T, total herbivores (grey), for remaining letter and colour codes, see figure 1. Rows represent separate model fits, coloured bars indicate that the predictor was included in a particular model and the height of each row adjusted to the cumulative Akaike weight, expressed as a proportion of all fitted models. I, model intercept term; ISL. TYPE, island type; CHL, chlorophyll a; COMP, reef complexity; CORAL, hard coral cover; HUM, human population density; PISC., piscivore biomass; TEMP, sea-surface temperature; AREA, area of habitat; WAVES, wave energy. (Online version in colour.)

Major drivers of this spatial variation in total herbivorous fish biomass were identified as reef complexity, hard coral cover, and human population density (electronic supplementary material, table S5). The original smoothers fitted to the functional group and total herbivore biomass values are in the electronic supplementary material, figure S5. Total herbivore biomass plateaued at intermediate complexity, decreased at highest coral cover, and continually decreased with human population density (electronic supplementary material, figure S5). The best-fit model that contained these three biological variables had high explanatory power and stability (approx. 69% variability explained in total herbivore biomass, 94% jack-knife stability; electronic supplementary material, table S5). When functional groups were modelled individually, the top candidate models showed similar stability. Specifically, the dominant predictors identified from the variable importance (vi) estimates from the top candidate model of the entire dataset matched those identified from the jack-knifing method (electronic supplementary material, table S5). The amount of variance explained by the top-ranking models of herbivore biomass for each functional group (figure 2) was as follows: browsers (84%); detritivores (84%); grazers (73%); scrapers/small excavators (36%); and large excavators/bioeroders (59%; electronic supplementary material, figure S5).

**Figure 3.**
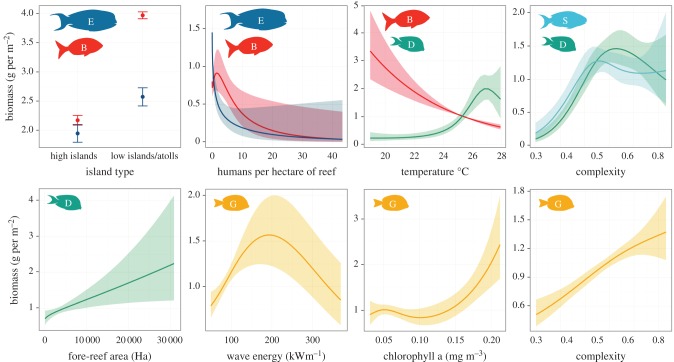
Predicted biomass and 95% confidence limits of functional groups by island type and along human and biophysical gradients: human population density; sea-surface temperature, habitat complexity; wave energy, area of fore reef and chlorophyll a. Functional groups are indicated by colour code and letter (figure 1).

The relationship between the top predictor terms and herbivore biomass was distinct for different functional groups. Biomass of large excavators/bioeroders (all parrotfishes more than 35 cm in total length) and browsers was significantly greater at low islands/atolls when compared with high islands (figure 3 and electronic supplementary material, table S5). These were also the only functional groups for which human population density was a strong predictor of biomass (figure 3 and electronic supplementary material, table S5), with both groups declining rapidly from low-to-mid human population density.

The dominant drivers of variability in browsers, detritivores, grazers, and scrapers/small excavators were biophysical. These data showed that reefs in warmer waters have lower browser biomass and greater detritivore biomass and species richness (figure 3 and electronic supplementary material, table S5). Increased detritivore, grazer and scraper/small excavator biomass was evident from low-to-mid habitat complexity. The biomass of grazers continued to increase at high complexity locations, whereas for detritivores and scrapers/small excavators the biomass either plateaued or was reduced at high complexity (figure 3). Locations with a larger amount of fore-reef habitat had greater biomass of detritivores, whereas areas with intermediate wave energy and high chlorophyll a had increased grazer biomass (figure 3 and electronic supplementary material, table S5).

## 4. Discussion

Our results are consistent with the growing understanding that regional variability in the biophysical attributes of coral reef ecosystems acts to determine ecological state independent of local human impacts [11,61,62]. Specifically, our findings confirm clear anthropogenic impacts to herbivorous fishes across the Pacific, but importantly also show that (i) effects are functional-group-specific, and (ii) the biophysical attributes of reefs, especially SST and large-scale geomorphological habitat complexity also drive herbivorous coral reef fish assemblage states. Prior to this study, quantitative evidence for anthropogenic impacts on herbivorous fishes, while simultaneously accounting for large-scale natural variability in fish assemblages, has been sparse [8,30,31]. To the best of our knowledge, this is the first ocean basin-scale study quantifying the relative effects of human versus natural biophysical drivers of herbivorous fish functional group biomass.

In the absence of fisheries-dependent data on subsistence, recreational and commercial take, human density, and distance to market have proven to be useful proxies for the influence of humans on coral reef fishes [11,63,64]. Our results show a steep and rapid decline in the biomass of large excavators and browsers with increasing human population density. This pattern is consistent with other global and regional assessments documenting the negative effect of fishing on herbivores [27,28]. Herbivorous fishes, in particular large excavating parrotfishes, and browsing surgeonfishes, are highly desired fisheries targets in the Pacific [65–68]. Our results demonstrate the sensitivity of populations of these large herbivores to fishing mortality, presumably owing to their large maximum body size and for some species, late age at maturity and the disproportionate contribution of large old females to population replenishment [65,69–72]. The vulnerability of these two functional groups to human impacts is particularly important as they contribute disproportionately to reef processes [50,73,74].

Herbivores vary in richness, abundance, and biomass by island geomorphology [8,31]. Our results show approximately 24–45% greater biomass of large-excavating and browsing fishes at low-lying islands (carbonate) and atolls, compared with high islands (basalt). There was no evidence for an island-type effect for the remaining functional groups, although consistent with a previous study [8], we found that the biomass of detritivores (all acanthurids) was positively associated with reef area. It may be that this island-type difference in biomass is driven by differential species-specific habitat requirements. For example, lagoonal habitat for settlement or nursery areas [75] is only present within atoll systems. The implications of our analyses are that large-scale habitat differences should be considered before comparing herbivorous fish assemblages across different island types.

Here, we found no consistent relationship between the biomass of different herbivore functional groups and the cover of hard corals, but still an overall relationship between coral cover and total herbivore biomass. Our results suggest that in areas of coral cover greater than 22–24% the total herbivore assemblage will tend to be reduced in biomass, whereas the biomass of grazers, detritivores, and scrapers/small excavators increases with habitat complexity, with peak biomass for scrapers and detritivores at intermediate complexity. Previously, a nonlinear association between coral species richness and fish community abundance has been shown [76], as has a reduction in abundances of small- and medium-sized herbivores at low habitat complexity [77]. Taking these effects of complexity and coral cover together, it seems plausible that this reflects the opposing changes in the availability of refugia and food with coral cover. In general, high coral cover, and associated structural complexity, reduces the foraging efficiency of predators [77–79]. Furthermore, the availability of shelter reduces the energy that fishes must allocate to swimming in high flow water environments [34], giving them an energetic advantage. As such, more complex environments support higher fish abundances [80]. However, increases in coral cover are accompanied by concomitant decreases in cover of other benthic organisms, such as turf, endolithic and macroalgae [81]. These algal assemblages, and associated detritus, are the primary food sources for herbivores, and as such food availability may limit population size in areas of high coral cover. This notion is supported by several studies that have documented increases in the abundance and biomass of herbivorous fishes following extensive coral mortality and reduced structural complexity [82–84].

The increased biomass of grazers in the areas of moderate wave exposure and increased oceanic productivity could also be related to food availability. Both algal and detrital mass tends to decrease with increasing wave energy and the highest edible algal mass occurs at moderately exposed reefs [85]. The positive association between chlorophyll a and grazer biomass could be owing to greater food availability for grazing fishes, specifically nutrients and sinking detrital particles such as phytodetritus, faeces, or dead planktonic material [77]. If this were the case, then one might expect to see a similar effect on detritivore biomass, however, we did not. Instead, the dominant biophysical driver of variability in detritivore biomass was SST.

Notably, detritivores and browsers showed opposing responses to SST, with browser biomass being negatively and detritivore biomass positively related to SST. Similar decreases in the biomass of browsing fishes with decreasing latitude, and hence SST, are evident in both the Atlantic [25] and southern Pacific Ocean [86]. Temperature fundamentally constrains the metabolic processes of ectotherms, and various hypotheses have been proposed to explain how temperature might impact the performance and fitness of individuals [87]. For instance, the temperature–size rule predicts ectotherms to be smaller in warmer waters, owing to reduced mean body size, earlier maturation, and increased initial growth rates [88–90]. While the temperature-constraint hypothesis relates to the interacting effects of temperature and food quality on fish physiology [25,91]. Here, we found increased browser biomass in cooler waters and increased detritivore biomass in warmer waters. Whether these trends in the standing stock of these functional groups relate to larger individuals and/or intraspecific variability in life-history characteristics across the temperature gradients surveyed would require location-specific, age-based studies on individual species.

The different effect of temperature on these functional groups could also be a response to the very different dietary strategies of these fishes. Browsing acanthurids, such as *Naso unicornis* and kyphosids, are the only functional group that hindgut ferment, which allows these fish to gain energy from refractory fleshy macroalgal carbohydrates, including mannitol [92–95]. Macroalgae, the preferred food of browsers, is more abundant on reefs in cooler climes in the Pacific [61] and thus browser biomass may be tracking the availability of their target resource. It is difficult to ascertain the primary nutrient sources of detritivores that feed on the epilithic algal matrix (EAM) [96]. The EAM contains a mixture of filamentous algal turfs, cyanobacteria, macroalgal spores, microalgae (diatoms and dinoflagelletes), heterotrophic bacteria, sediment, and organic detritus [97]. Stomach content analyses of the detritivore *Ctenochaetus striatus* reveal large amounts of loose plant cells, sediment, and algal filaments while the composition of short-chain fatty acids in *Ctenochaetus striatus* and *Ctenochaetus strigosus* guts are indicative of a diet of diatoms and bacteria [51,98]. Whether detritivorous fish biomass tracks spatial variability in the abundance and production of their target resource remains to be established.

## 5. Conclusion

Our findings highlight that coral reefs' biophysical setting strongly determine their carrying capacity and community composition of herbivorous reef fishes. Human impacts are superimposed over the backdrop of these naturally occurring drivers. Herbivore-focused management interventions are likely to become more widely implemented owing to the perception that greater herbivore biomass promotes reef resilience. Our results show large natural differences in the capacity of individual reefs to support herbivore populations and therefore, it is unlikely that all reefs will respond similarly to particular interventions, such as prohibition of fishing. Moreover, our results show that herbivore functional groups respond in different ways along gradients of those natural biophysical drivers. Locally appropriate management targets for herbivore functional group biomass must therefore factor in the natural bounds set by the reef's biophysical setting.

## Supplementary Material

Summary of sampling survey effort; Herbivorous fish functional classification; Predictors terms, estimating anthropogenic impacts and modeling; Herbivore species richness and biomass by region; Summary of the best performing GAMMs (all models with weight > 0.05) with smoother graphs.

## Data accessibility

All raw data collected for the Pacific Reef Assessment and Monitoring Programme are available upon request (email: nmfs.pic.credinfo@noaa.gov). All data used within the paper are available at https://github.com/adelheenan/ProcB_herbivores.
