# Supplementary material for: Natural bounds on herbivorous coral reef fishes
Source: Proc Biol Sci. 2016 Nov 30;283(1843):20161716. doi: 10.1098/rspb.2016.1716 (PMC5136584; doi:10.1098/rspb.2016.1716)
Supplement: Summary of sampling survey effort; Herbivorous fish functional classification; Predictors terms, estimating anthropogenic impacts and modeling; Herbivore species richness and biomass by region; Summary of the best performing GAMMs (all models with weight > 0.05) with smoother graphs. [file rspb20161716supp1.pdf]

Electronic supplementary material (ESM) from "Natural bounds on herbivorous coral reef fishes"

Adel Heenan, Andrew S. Hoey, Gareth J. Williams, Ivor D. Williams

Prepared for submission to the Proceedings of the Royal Society: Biological Sciences.

ESM. S1. Summary of sampling survey effort

Table S1. Number of replicate stationary point count and towed-diver surveys per island / atoll or island group with regional subtotals. Island latitude (lat) and longitude (long) and area of reef (km^2 of hard-bottom habitat within 0-30 m depth contour) are also displayed. Unless otherwise specified in the island code, islands are referred to in full name. Regions refer to the provinces identified in the Marine Ecoregions of the World [1], C.Polynesia = Central Polynesia, Marshall Is. = Marshall, Gilbert and Ellis Islands, Trop.NW.Pacific = Tropical Northwestern Pacific.

Region	Island	Code	Lat.	Long.	Area	SPC (n)	SPC region total (n)	Tow (n)	Tow region (n) total
	Kingman		6.4	-162.38	37.21	79		38	
	Palmyra		5.88	-162.09	42.13	158		65	
	Howland		0.8	-176.62	1.73	90		27	
	Baker		0.2	-176.48	3.9	81		24	
	Jarvis		-0.37	-160	3.66	134		23	
C.Polynesia	Swains		-11.06	-171.08	2.81	94	**1308**	25	**398**
	Ofu & Olosega	O&O	-14.17	-169.65	10.55	112		34	
	Tau		-14.24	-169.47	10.03	92		39	
	Tutuila		-14.3	-170.7	48.88	374		100	
	Rose		-14.55	-168.16	5.64	94		23	
	Kure		28.42	-178.33	36.99	37		8	
	Pearl & Hermes	P&H	27.86	-175.85	178.12	69		21	
	Lisianski		26.01	-173.95	309.55	104		10	
	French Frigate	FFS	23.79	-166.21	277.97	48		18	
	Kauai		22.09	-159.57	184.21	82		22	
Hawaii	Niihau		21.9	-160.15	94.02	90	**1214**	9	**218**
	Oahu		21.49	-158	306.4	171		14	
	Molokai		21.14	-157.09	144.95	147		11	
	Lanai		20.82	-156.92	36.03	88		10	
	Maui		20.82	-156.4	117.72	140		24	
	Hawaii		19.53	-155.42	161.96	198		37	
	Johnston		16.74	-169.52	94.1	40		34	
Marshall Is.	Wake		19.3	166.62	12.82	75	**75**	24	**24**
	Farallon de Pajaros	FDP	20.55	144.89	1.38	23		6	
	Maug		20.02	145.22	3.14	70		17	
	Asuncion		19.69	145.4	2.49	41		11	
	Agrihan		18.76	145.66	8.51	20		10	
	Pagan		18.11	145.76	15.13	72		25	
Trop. NW.Pacific	Alamagan-Guguan-Sarigan	AGS	17.2	145.81	2.48	57	**712**	21	**221**
	Saipan		15.19	145.75	48.47	78		30	
	Tinian		14.99	145.63	14.14	38		21	
	Aguijan		14.85	145.55	4.06	23		7	
	Rota		14.16	145.21	13.31	52		19	
	Guam		13.46	144.79	71.01	238		54	

ESM.S2. Herbivorous fish functional classification

The functional importance of herbivores centres on species-specific feeding behaviour, and the impact their feeding has on coral-algal dynamics. Here, we use a functional classification scheme for larger roving herbivorous fishes (primarily the Acanthuridae, Scarinae, Siganidae, and Kyphosidae) that is widely accepted [2]. This classification differentiates functional groups based on a combination of their diet, morphology of the feeding apparatus, feeding behavior, and impact on benthic communities. We do not consider smaller territorial herbivorous species (i.e., Pomacentridae) as they have a markedly different impact on the benthos than the larger roving species, typically increasing the biomass of algae within their territories (e.g. [3,4]).

Herbivorous fishes may be broadly classified into those that feed on fleshy or erect macroalgae, and those that feed on the epilithic algal matrix (EAM). The EAM is a combination of algal turfs, macroalgal propagules, detrital aggregates, sediment and associated microbial communities, and is typically <10 mm in height [1-3]. Those species that feed on fleshy, or erect macroalgae, that is generally > 10 mm, are termed 'browsers'. Those species that feed on EAM covered substrata may be further divided based on the materials they are targeting or ingest while feeding. 'Grazers' refer to those species that crop the upper portions of algae leaving the basal portions intact [5–7]. In contrast, scraping and excavating parrotfishes remove parts of the underlying substrata together with the EAM when feeding, and in doing so clear space for the settlement of benthic organisms (including corals). The parrotfishes may be further divided into 'scrapers/small excavators' and 'large excavators/bioeroders' based on the amount of the substratum that is removed during feeding; scrapers and small excavating species leave shallow (<1mm) bite scars, large excavators take deeper bites and remove greater quantities of substrata with each bite. Finally, the 'detritivores' brush the epithlic algal matrix with highly specialized mouthparts and in doing may consume small filamentous turf algae, along with large amounts of unidentified organic matter and bacteria [8–10]. Collectively, these groups that feed on the EAM maintain algal communities in a cropped and highly productive state [2,11,12], and in doing so have been implicated in preventing the establishment of macroalgae. In addition, the grazers and scrapers and excavators may facilitate the settlement, survival and growth of crustose coralline algae and coral [13,14]. The scraping and excavating parrotfish are further divided by size. Large excavators/bioeroders can act as major agents of bioerosion, consuming greater quantities of the reef matrix than their smaller counterparts. The deep bites by large parrotfishes may be of increased functional importance in terms of impeding macroalgal dominance, opening up new settlement sites for coral recruitment and promoting asexual reproduction of corals by creating and dispersing coral fragments [2,15].

Some herbivorous species also exhibit ontogenetic shifts in diet, or differential functional impacts with body size and this was accounted for in our functional classifications using the following size thresholds. Several species of *Naso* undergo ontogenetic shifts in diet, feeding on fleshy macroalgae as juveniles and gelatinous zooplankton as adults [2]. As such, we only included juvenile (< 20 cm TL) *Naso annulatus* and *Naso brevirostris* as browsers, the adults of these species were excluded from our classifications of herbivores [2]. Similarly the amount of material removed by parrotfishes when feeding has been related to their body size, both among and within species. For example, small individuals of excavating genera (i.e., *Chlorurus*, *Cetoscarus* and *Bolbometopon*) essentially function as scrapers, and conversely large individuals of some scraping species may function as excavators. To account for this variation in feeding impact we classified *Hipposcarus*, all *Scarus* (except *S. rubroviolcaeus* > 35cm TL), all small excavating species (*C. spilurus* and *C. japanensis*) and small individuals of

larger excavating species (*C. frontalis* and *C. microrhinos* ≤ 20cm TL, and *Cetoscarus* and *C. perspicillatus* ≤ 35cm TL) as scrapers/small excavators. Larger excavators/bioeroders included *C. frontalis* and *C. microrhinos* > 20cm TL, and *Cetoscarus*, *C. perspicillatus* and *S. rubroviolaceus* > 35cm TL).

ESM S3. Predictors terms, estimating anthropogenic impacts and modeling

Table S3.1 Island level predictor terms used in the modeling process. With the exception of piscivore biomass, the biotic predictors (reef complexity, hard coral cover) were island-scale averages calculated from the site level survey data. Piscivore biomass was calculated from the towed-diver survey method, a method that is better optimized for large roving fishes. Our estimates of total area of forereef were obtained from habitat maps maintained by the Pacific Reef Assessment and Monitoring Program, and the density of humans derived for the number of people per island (from the US Population Census) divided by the area of fore-reef. The environmental predictors (sea surface temperature, wave energy and chlorophyll-a were all taken from [16].

**Predictor**	**Label**	**Details**
Island type	IS.TYPE	High or low lying island / atoll
Productivity	CHL_	Oceanic productivity climatological mean of (chlorophyll-a mg m^{-3}) between July 2002-May 2011
Complexity	COMP	Mean substrate mean within the fish survey area, pooled at the island level
Hard coral cover	CORAL	Mean % coral cover within the fish survey area, pooled at the island level
Human impact	HUM	Square root transformed number of humans resident per island (from the US 2010 census) divided by area of fore-reef
Piscivore biomass	PISCI	Mean piscivore biomass g m^{-2} per island from towed diver surveys.
Sea surface temperature	TEMP	Lower climatological mean of sea surface temperature, i.e. the average of mean temperature in the coldest month of each year between 1985 and 2009
Reef area	AREA	Area of fore-reef (< 30 m hard-bottom habitat) per island from habitat maps maintained for Pacific RAMP
Wave exposure	WAVES	Climatological mean of wave energy (kW m^{-1}) between 1997 and 2010

ESM.3. Quantifying anthropogenic impacts on coral reef fishes

Because there are rarely high quality local data on fishery extraction or effort, studies on human impacts to coral reef fishes have generally relied on proxies, such as human population or human population per unit of reef [17–21]. However, a number of recent studies suggest that fishery impacts are better represented by metrics, such as distance and travel to market and/or human population centers [22–25].

For this study, we chose to use human population density per unit of forereef habitat as our proxy for anthropogenic impacts on the fish assemblage, which has been shown to be useful for these reefs [19,21]. While we recognise the utility of distance and time metrics for global scale studies and for locations where the presence of centralised markets and/or harbours around population centers drive much of the fishing effort [22,24–26], they are unlikely to be suited to several of the locations included in our study. In Hawaii, the Commonwealth of the Northern Mariana Islands and in American Samoa, the large majority of reef fish catch is taken for recreational or subsistence purposes, and is therefore either sold informally or retained for consumption, gifting or exchange [27–29]. Furthermore, the reef fishery in Hawaii is dominated by shore-based rather than boat-based fishing, which weakens the link between catch or effort and the location of population centers and harbours. Additionally, for the islands included in this study, our estimate of humans per hectare of reef is strongly correlated to distance to nearest provincial capital (see figure S3), and therefore it seems unlikely that the choice of human-impact-proxy would substantially alter the main findings reported here.

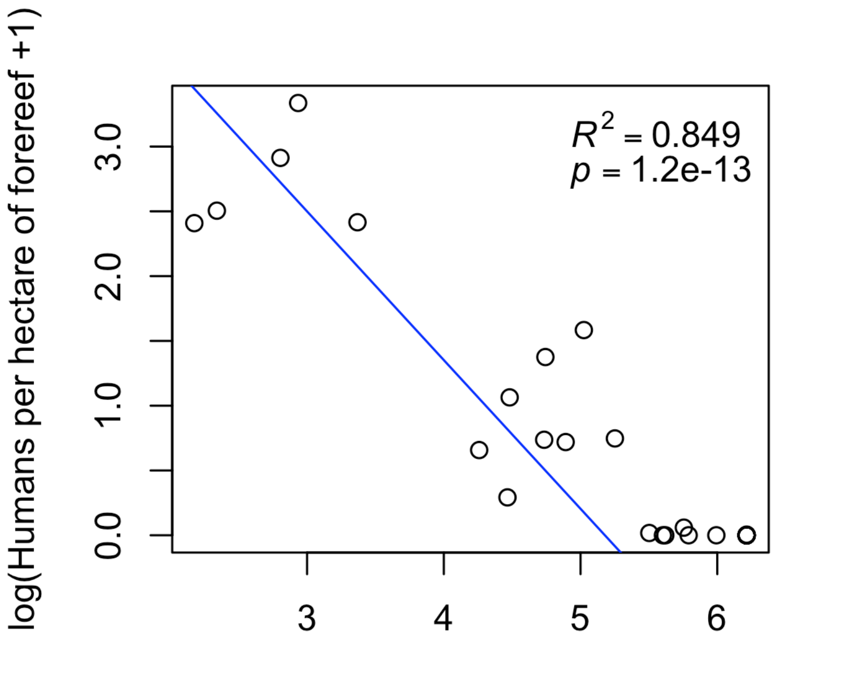

Figure S3. The number of humans per hectare of reef (log +1 transformed) decreases linearly with the distance of a reef location to the nearest provincial capital (log +1 transformed) (R^2 = 0.85, p = < 0.01). Distances greater than 500 km at capped at this value.

ESM.S3. Model fitting method and analyses

The herbivorous fish assemblage in any one location could be characterized by abundance and biomass of particular species as well as functional group biomass and richness. A positive association between species richness and total fish biomass exists at a large, global scale [30] and in the absence of human impacts, any differences in fish biomass and richness across the islands we survey is a product of the interaction between both ecological and evolutionary processes.

Here, we wanted to explore the relationship between biomass and functional group diversity. To do this, we fitted linear mixed effects models. There are clear regional differences in the species richness of these herbivore functional groups and a general positive association between the two for some functional groups (electronic supplementary material S4). We then proceeded to focus on modeling fish biomass to investigate the influence of humans and biophysical drivers of the ecological status of herbivore assemblages. We focused on fish biomass because 1) biomass is more actionable on a local management scale and 2) we wanted our analysis to relate to broader ongoing efforts that have used fish biomass as a proxy human impacts on coral reef assemblages in global analyses [31,32].

Prior to calculating island level mean estimates of fish species biomass, we inspected site level species biomass estimates for outlying observations. We defined outliers are those > 97.5% of the interquartile range. The majority of outliers were due to random encounters with extremely high counts of individual species, such as large aggregations of *Acanthurus achilles* or *Acanthurus triostegus*. Encounters with such aggregations introduce large variability in the data, which is likely not representative of the species' biomass across the reef areas surveyed. To reduce the influence of those encounters, we capped outliers at the 97.5% biomass quantile for each species.

Prior to model fitting, we examined the distributions of mean fish biomass per functional group and total herbivore biomass per island, and, as they tended to be left skewed and are bounded by zero, we elected to fit models using a Gamma error structure with a log-link function. All of the predictor variables were standardised (mean centered, and divided by their standard deviations) prior to model fitting. The transformed values used were therefore unit-less, centered on zero, with a variance of one.

To check for co-linearity of explanatory variables, we calculated Pearson's correlation coefficients and variance inflation factors among all combinations of predictor variables. With the exception of SST and wave energy, all variance inflation factor values were less than 3, indicating that co-linearity was acceptably low [33]. For each response variable, we therefore identified and excluded the weakest predictor between SST and wave-energy. Specifically, we fitted GAMMs for all possible combinations of the full set of predictor variables using the *UGamm* wrapper function that allows for mixed effects model structures, in combination with the *dredge* function in the *MuMIn* package [34]. We then calculated Akaike's Information Criterion, corrected for small sample size (AICc), and relative importance weights (w_i) of each model. Sum of all model weights is 1, and predictor variables that tend to feature in models with high likelihood of being the best model have high variable importance (i.e. high Σw_i). For each predictor variable, we picked whichever of SST or wave-energy had highest variable importance, and dropped the other prior to proceeding with the analysis. For all response variables, there were either clear differences in the variable importance (one of either SST or wave

energy was much higher than the other) or neither variable had high importance (Table S3.2).

All analysis presented in the main body of the study was therefore conducted with models containing either SST or wave energy.

Table. S3.2. Variable importance from full model sets including both SST and wave energy. Predictor variable included in final model sets highlighted in bold.

**Response Variable**	**Variable Importance**	
	**SST**	**Wave energy**
All herbivores	**7**	3
Browsers	**95**	13
Detritivores	**93**	19
Grazers	4	**94**
Scrapers/sm. excavators	**24**	18
Large excavators/bioeroders	**38**	9

ESM.S4. Herbivore species richness and biomass by region.

Table S4. Proportion of sightings per species in each functional group. Main = Main Hawaiian Islands, (populated) NW = Northwest Hawaiian Islands (unpopulated), Trop NW Pac = Tropical Northwest Pacific (South = populated, North = unpopulated). Colours relate to the coding used throughout the manuscript (Browsers = red, Detritivores = green, Grazers = yellow, Scrapers/excavators (small)= light blue, Excavators/bioeroders (large) = dark blue). Within each functional group proportions are conditional shaded, where the higher the proportion, the darker the shade.

**Browsers**	**Hawaii**		**Marshall**	**Trop NW Pac**		**Central Polynesia**		
	**Main**	**NW**	**Wake**	**South**	**North**	**Phoenix**	**Line**	**Samoa**
Calotomus carolinus	0.03	0.01	<0.01	0.06	<0.01	0.09	0.26	0.11
Calotomus zonarchus	<0.01	0.02	0	0	0	0.01	<0.01	<0.01
Kyphosus cinerascens	<0.01	<0.01	0	0	<0.01	0	0.06	0.02
Kyphosus hawaiiensis	0	<0.01	0	0	0	0	0	0
Kyphosus sandwicensis	<0.01	0.02	0	0	0	0	0	0
Kyphosus vaigiensis	<0.01	0	0	<0.01	<0.01	0	0	0
Naso annulatus	<0.01	0	0	0	0	0	0	0
Naso brachycentron	0	0	0	<0.01	0	0	0	<0.01
Naso brevirostris	0.1	0.05	<0.01	<0.01	<0.01	<0.01	0	<0.01
Naso lituratus	0.49	0.05	0.14	0.87	0.58	0.58	0.48	0.76
Naso tonganus	0	0	0	0.02	<0.01	0	0	0.01
Naso unicornis	0.12	0.35	<0.01	0.03	<0.01	0.04	0.09	0.02
Platax teira	0	0	0	<0.01	0	<0.01	0	<0.01
Kyphosus sp.	0.25	0.49	0.86	0.02	0.4	0.26	0.11	0.07

**Detritivores**	**Hawaii**		**Marshall**	**Trop NW Pac**		**Central Polynesia**		
	**Main**	**NW**	**Wake**	**South**	**North**	**Phoenix**	**Line**	**Samoa**
Ctenochaetus binotatus	0	0	0	0.21	0.09	<0.01	<0.01	0.02
Ctenochaetus cyanocheilus	0	0	0.52	0.03	0.4	0.46	0.25	0.22
Ctenochaetus flavicauda	0	0	0	0	0	0.04	0.02	<0.01
Ctenochaetus hawaiiensis	0.03	<0.01	0.1	0.01	0.09	0.03	0.02	<0.01
Ctenochaetus marginatus	0	0	0	0	0	0.41	0.33	0.00
Ctenochaetus striatus	0	0	0.38	0.75	0.42	0.05	0.37	0.76
Ctenochaetus strigosus	0.97	1	0	0	0	0	0	0.00

Grazers	Hawaii		Marshall	Trop NW Pac		Central Polynesia		
	Main	NW	Wake	South	North	Phoenix	Line	Samoa
Abudefduf sordidus	<0.01	<0.01	<0.01	<0.01	<0.01	<0.01	<0.01	<0.01
Acanthurus achilles	0.02	<0.01	0.02	<0.01	0	0.04	0.01	0.03
Acanthurus blochii	0.01	0.02	<0.01	<0.01	<0.01	<0.01	<0.01	<0.01
Acanthurus dussumieri	0.01	<0.01	0	0	<0.01	0	<0.01	0
Acanthurus guttatus	<0.01	0	<0.01	<0.01	<0.01	<0.01	<0.01	0.01
Acanthurus leucocheilus	0	0	0	0	0	<0.01	<0.01	<0.01
Acanthurus leucopareius	0.12	0.09	0	0	0.06	0	<0.01	0
Acanthurus lineatus	0	<0.01	0	0.06	0.06	0.03	0.03	0.08
Acanthurus maculiceps	0	<0.01	0	0	0	<0.01	<0.01	<0.01
Acanthurus nigricans	<0.01	<0.01	<0.01	0.07	0.11	0.23	0.35	0.25
Acanthurus nigricauda	0	0	<0.01	<0.01	<0.01	<0.01	<0.01	<0.01
Acanthurus nigrofuscus	0.41	0.1	0.15	0.53	0.21	<0.01	0.01	0.27
Acanthurus nigroris	0.1	0.17	0.38	<0.01	<0.01	0.03	0.02	0.01
Acanthurus olivaceus	0.06	0.08	<0.01	0.04	0.02	0.06	0.02	0.02
Acanthurus pyroferus	0	0	0	0.02	0.02	<0.01	<0.01	<0.01
Acanthurus triostegus	0.05	0.3	0.07	0.01	<0.01	0.17	0.17	0.01
Acanthurus xanthopterus	<0.01	<0.01	0	<0.01	<0.01	<0.01	<0.01	<0.01
Centropyge bicolor	0	0	0	0	0	<0.01	0	<0.01
Centropyge bispinosa	0	0	0	<0.01	<0.01	<0.01	0	0.08
Centropyge fisheri	<0.01	<0.01	0	0	<0.01	0	0	<0.01
Centropyge flavissima	0	<0.01	0.15	0.06	0.14	0.15	0.2	0.14
Centropyge heraldi	0	0	0	0.04	0.01	0	0	0.01
Centropyge loricula	0.02	<0.01	0.13	<0.01	0	0.25	0.15	0.02
Centropyge potteri	0.03	0.16	0	0	0	0	0	0
Centropyge shepardi	0	0	0	0.13	0.25	0	0	0
Centropyge vrolikii	0	0	0	<0.01	0	<0.01	0	0
Siganus argenteus	0	0	0	<0.01	0	0	0	<0.01
Siganus punctatus	0	0	0	<0.01	0	0	0	0
Siganus spinus	0	0	0	<0.01	0	0	0	0
Zebrasoma flavescens	0.16	0.03	0.07	0.02	0.08	0	<0.01	<0.01
Zebrasoma rostratum	0	0	0	0	0	<0.01	0.01	<0.01
Zebrasoma scopas	0	0	0	<0.01	0	0.03	<0.01	0.03
Zebrasoma veliferum	<0.01	0.02	<0.01	<0.01	<0.01	<0.01	<0.01	<0.01

Scrapers /small excavators	Hawaii		Marshall	Trop NW Pac		Central Polynesia		
	Main	NW	Wake	South	North	Phoenix	Line	Samoa
Cetoscarus ocellatus	0	0	0	<0.01	<0.01	0	0	<0.01
Chlorurus frontalis	0	0	0	0.01	<0.01	0	0	<0.01
Chlorurus japanensis	0	0	0	0	0	0	0	0.13
Chlorurus microrhinos	0	0	0	<0.01	<0.01	0	<0.01	<0.01
Chlorurus perspicillatus	0.02	0.14	0	0	0	0	0	0
Chlorurus spilurus	0.57	0.34	0.45	0.56	0.02	0.02	0.47	0.46
Hipposcarus longiceps	0	0	0	<0.01	0	0	<0.01	0
Scarus altipinnis	0	0	0	0.03	0	0	<0.01	<0.01
Scarus dimidiatus	0	0	0	<0.01	0	0	0	<0.01
Scarus dubius	0.02	0.36	0	0	0	0	0	0
Scarus festivus	0	0	0	<0.01	0	<0.01	<0.01	<0.01
Scarus forsteni	0	0	0.44	0.06	0.52	0.06	0.03	0.09
Scarus frenatus	0	0	0	<0.01	<0.01	0.23	0.17	0.04
Scarus fuscocaudalis	0	0	0	<0.01	0	0	0	0
Scarus ghobban	0	0	0.01	<0.01	<0.01	0	0.01	<0.01
Scarus globiceps	0	0	<0.01	0.01	0	0	0.01	0.02
Scarus niger	0	0	0	0	0	0.04	<0.01	<0.01
Scarus oviceps	0	0	0.01	<0.01	0.06	0.02	0.07	0.04
Scarus psittacus	0.32	0.07	0.08	0.18	0.02	<0.01	0.02	0.07
Scarus rubroviolaceus	0.06	0.01	<0.01	0.01	0.37	0.29	0.06	0.03
Scarus schlegeli	0	0	0	0.07	<0.01	0	0	0.02
Scarus spinus	0	0	0	0	0	0.01	<0.01	0.02
Scarus tricolor	0	0	0	0	0	0.28	0.09	<0.01
Scarus xanthopleura	0	0	0	0	0	0.03	0	<0.01

Large excavators / bioeroders	Hawaii		Marshall	Trop NW Pac		Central Polynesia		
	Main	NW	Wake	South	North	Phoenix	Line	Samoa
*Bolbometopon muricatum**	0	0	0	0	0	0	0	0
Cetoscarus ocellatus	0	0	0	0.03	<0.01	0	0	0.02
Chlorurus frontalis	0	0	0.85	0.61	0.06	0	0.1	0.48
Chlorurus microrhinos	0	0	0.15	0.06	0.19	0.16	0.61	0.35
Chlorurus perspicillatus	0.26	0.97	0	0	0	0	0	0
Scarus rubroviolaceus	0.74	0.03	0	0.29	0.75	0.84	0.29	0.14

* The largest of all parrotfishes is the giant bumphead parrotfish *Bolbometopon muricatum* [35]. This is a wide-ranging species that occurs throughout the Indo Pacific, including all regions surveyed in this study, except Hawaii [35]. *B.muricatum* is considered functionally unique, in its role as a major bioeroder and coral predator, that opens up bare substrate for coral settlement and facilitates the dispersal of coral fragments [15,36,37]. It is noteworthy then, that we failed to detect this species at the majority of the islands/atolls surveyed. Over the approximately 13,600 km^2 of total reef area surveyed within it's range, we only recorded 34 individuals on transect, at Wake

Atoll (n=34) and Guam (n=3), all of which were seen on the towed-diver method. In fact, this species has been recorded in low densities on U.S. and U.S. affiliated reefs since 2000 [38]. The sampling design, level of effort and frequency of the monitoring data utilized here is not optimized to detect species-specific trends [39]. This limits our ability for inference, particularly for this large, rare species that is often habitat specific, patchily distributed and subject to behaviorally driven short-term temporal variation [37,40]. Whether the absence of sightings of *B.muricatum* in these data substantiates evidence of a further reduction of this threatened species (IUCN Red List; Vulnerable) warrants further investigation [35,41,42].

ESM S4. Modeling output for richness and biomass

Mixed-effect models of biomass and richness per functional group with region as a random effect were implemented using the "glmer" function from the "lme4" library [43] in R v.3.2.5 (R Development Core Team 2016). The minimal models (identified by deleting non-significant interactions and main effects) are presented in Table S4. Significant differences were evaluated with maximum likelihood ratio tests (χ^2, $p < 0.05$).

The biomass and richness of herbivorous fishes was positively related in large excavators/bioeroders, scrapers and small excavators and detritivores (Figure S4, Table S4). The biomass of large excavators/bioeroders and scrapers and small excavators is higher at un-populated islands, this is also true for detritivores but the slope of this relationship is steeper for populated islands (Figure S4, Table S4). For all other groups (browsers and grazers) there was no significant relationship between biomass and richness (Table S4).

Figure S4. Relationship between herbivorous fish functional group biomass and richness. Data are coded by region, C.Polynesia = Central Polynesia, Trop.NW.Pacific = Tropical Northwestern Pacific.

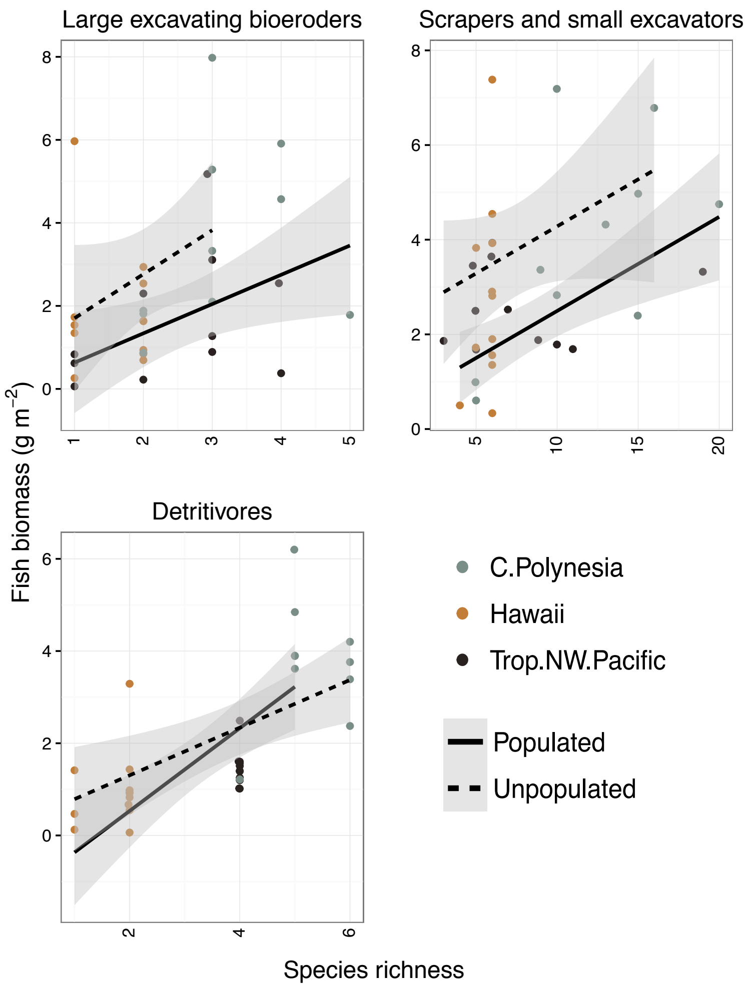

Table S4. The minimal models for biomass and species richness of each functional group (F.G.). Non-significant interactions and main effects were deleted from the model (P> 0.05). B = browsers, D = Detritivores, G = Grazers, S = scrapers and small excavators, E = Large excavators and bioeroders.

F.G. Biomass	Term	Co-efficient	SE	t-value	p-value	Sig-code
**B**	(Intercept)	1.83	0.96	1.90	0.06	
	Pop	2.06	0.75	2.73	0.01	***
**D**	(Intercept)	-1.76	1.32	-1.33	0.18	
	Richness	1.06	0.34	3.10	0.00	***
	Pop	2.05	0.82	2.51	0.01	***
	Richness*Pop	-0.56	0.22	-2.51	0.01	***
**G**	(Intercept)	6.04	2.45	2.47	0.01	***
	Richness	-0.18	0.13	-1.34	0.18	
**S**	(Intercept)	0.51	0.62	0.82	0.41	
	Richness	0.20	0.06	3.43	<0.01	***
	Pop	1.79	0.50	3.54	<0.01	***
**L**	(Intercept)	-0.32	0.97	-0.33	0.74	
	Richness	0.81	0.32	2.56	0.01	***
	Pop	1.47	0.62	2.38	0.02	**

ESM. S5. Summary of the best performing GAMMs (all models with weight > 0.05) with smoother graphs.

Table S5. For the biomass of each group, models with the greatest predictive power are presented in the first rows in light grey, predictors terms included in each model are signaled with a + sign, rows below this present a summary of the importance of each variable from model averaging and during the jack-knife sensitivity test. See Table S3 for definition of predictor terms. Columns aR^2 = adjusted R^2 (proportion of variance explained), df = degrees of freedom, Loglik = log likelihood, AICc= Akaike Information Criterion corrected for small sample size, d = difference in AICc relative to the top candidate model, w = Akaike weight, JK = jack knife sensitivity (% time the same top model was identified). Importance = sum total weight of all models containing each variable (high values indicate a high percentage of models containing that particular variable), JK importance mean and se = the percentage mean importance and standard error estimate obtained during the jack knife sensitivity test. The importance variables are conditional color coded, where the higher the importance metric, the darker shade of red. The dark greyed out columns refer to the exclusion of SST or wave energy during the model fitting process due to their co-linearity (see ESM 3 for full explanation).

RESPONSE	PREDICTOR TERMS									MODEL PERFORMANCE						
**Browsers**	**ISL.**	**CHL_**	**COMP**	**CORAL**	**HUM**	**PISCI**	**TEMP**	**AREA**	**WAVES**	**aR^2**	**df**	**logL**	**AICc**	**d**	**w**	**JK**
Model 1	+		+		+		+			0.84	10	-37.31	104.62	0.00	0.35	0.58
Model 2	+				+		+			0.79	8	-41.36	104.73	0.10	0.33	
Model 3	+	+			+		+			0.83	10	-37.84	105.68	1.05	0.21	
Model 4	+				+		+	+		0.82	10	-38.40	106.79	2.17	0.12	
Importance	86.40	21.69	30.96	1.41	99.16	1.89	98.48	17.15								
JK importance (mean)	81.64	21.42	28.87	1.82	97.66	3.76	95.82	19.78								
JK importance (se)	0.02	0.03	0.02	0.00	0.01	0.01	0.02	0.02								
**Detritivores**	**ISL.**	**CHL_**	**COMP**	**CORAL**	**HUM**	**PISCI**	**TEMP**	**AREA**	**WAVES**	**aR^2**	**df**	**logL**	**AICc**	**d**	**w**	**JK**
Model 1			+				+	+		0.84	9	-26.40	78.63	0.00	0.78	0.85
Model 2	+		+				+	+		0.85	10	-25.60	81.19	2.56	0.22	
Importance	26.10	3.18	99.99	5.75	1.40	1.43	92.72	84.10								
JK importance (mean)	29.01	3.93	99.96	8.60	1.46	1.75	87.43	71.58								
JK importance (se)	0.02	0.00	0.00	0.02	0.00	0.00	0.02	0.04								
**Grazers**	**ISL.**	**CHL_**	**COMP**	**CORAL**	**HUM**	**PISCI**	**TEMP**	**AREA**	**WAVES**	**aR^2**	**df**	**logL**	**AICc**	**d**	**w**	**JK**
Model 1		+	+	+					+	0.73	11	-45.72	126.01	0.00	0.51	0.79
Model 2		+	+						+	0.62	9	-50.74	127.31	1.30	0.27	
Model 3	[illegible]	[illegible]	[illegible]						[illegible]	[illegible]	[illegible]	[illegible]	[illegible]	[illegible]	[illegible]	

Scrapers/sm ex. (small)	ISL.	CHL_	COMP	CORAL	HUM	PISCI	TEMP	AREA	WAVES	aR²	df	logL	AICc	d	w	JK
Model 1	+		+							0.36	6	-55.84	126.91	0.00	0.43	0.79
Model 2			+							0.28	5	-57.73	127.68	0.78	0.29	
Model 3	+		+	+						0.43	8	-53.92	129.84	2.93	0.10	
Model 4			+				+	+		0.50	9	-52.02	129.87	2.97	0.10	
Model 5			+			+				0.36	7	-55.89	130.27	3.36	0.08	
Importance	52.01	6.48	91.37	15.33	4.20	12.12	18.27	18.09								
JK importance (mean)	49.05	6.15	86.77	18.91	5.06	15.03	18.14	20.88								
JK importance (se)	0.02	0.00	0.03	0.02	0.00	0.02	0.02	0.03								

Excavators/bioeroders (large)	ISL.	CHL_	COMP	CORAL	HUM	PISCI	TEMP	AREA	WAVES	aR²	df	logL	AICc	d	w	JK
Model 1	+				+		+			0.59	8	-45.23	112.47	0.00	0.31	0.58
Model 2	+				+			+		0.58	8	-45.55	113.10	0.63	0.23	
Model 3	+				+		+	+		0.67	10	-42.05	114.10	1.63	0.14	
Model 4					+					0.41	5	-51.06	114.35	1.88	0.12	
Model 5					+			+		0.51	7	-47.99	114.46	1.99	0.12	
Model 6	+				+					0.45	6	-49.92	115.07	2.60	0.08	
Importance	67.11	3.15	9.74	11.31	99.91	7.61	36.39	41.16								
JK importance (mean)	62.43	3.07	10.51	11.66	99.84	7.74	32.05	40.36								
JK importance (se)	0.02	0.00	0.01	0.01	0.00	0.01	0.03	0.03								

All herbivores	ISL.	CHL_	COMP	CORAL	HUM	PISCI	TEMP	AREA	WAVES	aR²	df	logL	AICc	d	w	JK
Model 1			+	+	+					0.69	9	-90.09	206.00	0.00	0.61	0.94
Model 2			+		+					0.59	7	-94.74	207.96	1.96	0.23	
Model 3	+		+	+	+					0.70	10	-89.29	208.59	2.59	0.17	
Importance	19.50	2.42	95.14	70.73	96.68	3.03	6.85	2.10								
JK importance (mean)	18.99	2.49	92.40	67.65	95.13	5.77	7.54	2.38								
JK importance (se)	0.01	0.00	0.01	0.03	0.00	0.01	0.01	0.00								

ESM Figure S5. Smoother of predictor variables retained in the highest ranked models for functional group and total herbivore biomass. Shaded areas display 95% confidence and red = browsers, green = detritivores, yellow = grazers, light blue = small scrapers/excavators, dark blue = large excavating bioeroders and grey = total herbivore biomass.

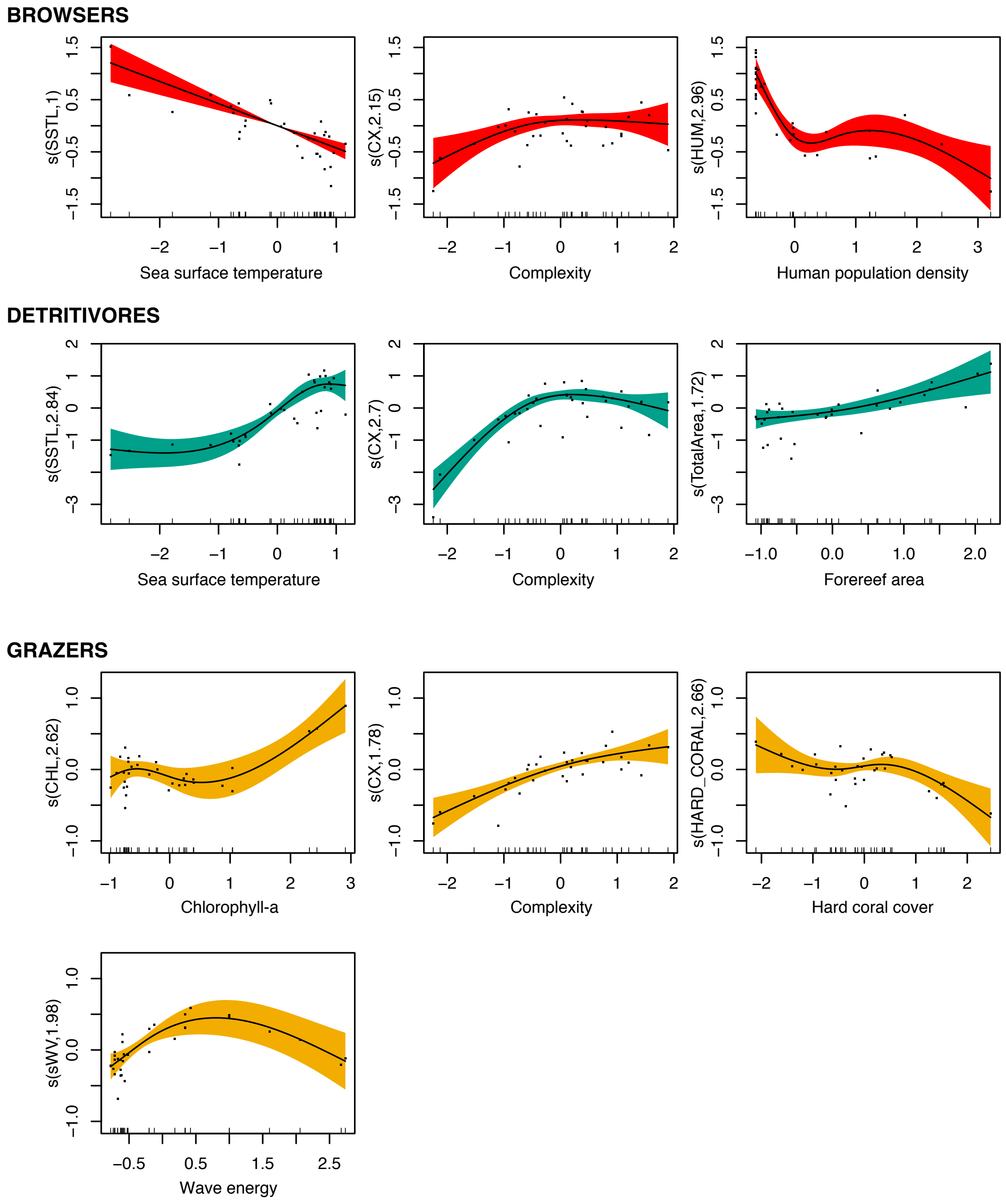

SCRAPERS AND SMALL EXCAVATORS

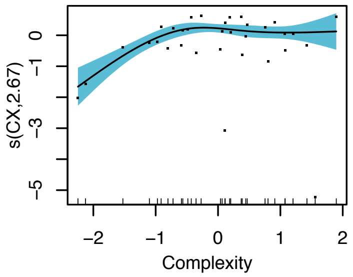

LARGE EXCAVATORS AND BIOERODERS

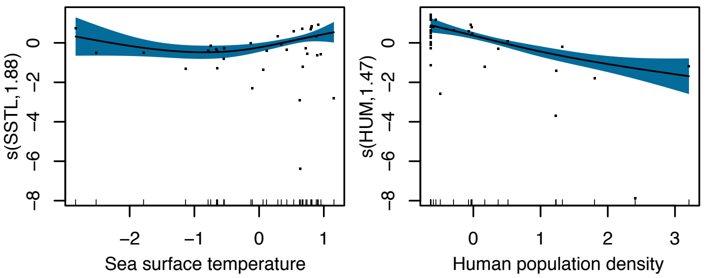

ALL HERBIVORES

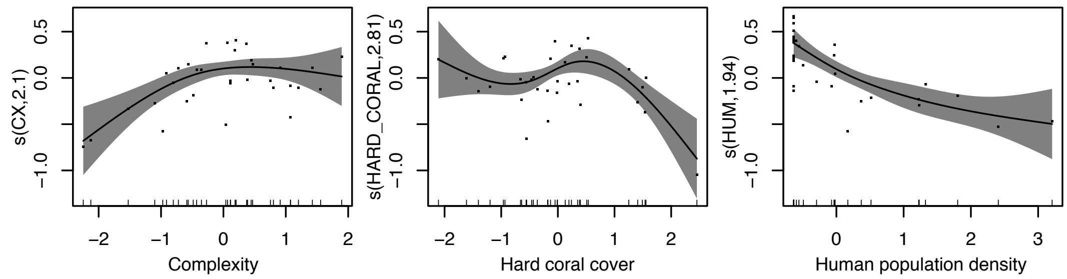

References

1. Spalding, M. D. et al. 2007 Marine ecoregions of the world: a bioregionalization of coastal and shelf areas. *Bioscience* **57**, 573. (doi:10.1641/B570707)

2. Green, A. L. & Bellwood, D. R. 2009 Monitoring functional groups of herbivorous reef fishes as indicators of coral reef resilience - a practical guide for coral reef managers in the Asia Pacific region. IUCN, Gland, Switzerland. 70 p. (ISBN: 978-2-8317-1169-0)

3. Ceccarelli, D., Jones, G. P. & Mccook, J. 2001 Territorial damselfishes as determinants of the structure of benthic communities on coral reefs. *Oceanogr. Mar. Biol. An Annu. Rev.* **39**, 355–389.

4. Hoey, A. S. & Bellwood, D. R. 2010 Damselfish territories as a refuge for macroalgae on coral reefs. *Coral Reefs* **29**, 107–118. (doi:10.1007/s00338-009-0567-8)

5. Hughes, T. P. et al. 2007 Phase shifts, herbivory, and the resilience of coral reefs to climate change. *Curr. Biol.* **17**, 360–5. (doi:10.1016/j.cub.2006.12.049)

6. Paddack, M. J., Cowen, R. K. & Sponaugle, S. 2006 Grazing pressure of herbivorous coral reef fishes on low coral-cover reefs. *Coral Reefs* **25**, 461–472. (doi:10.1007/s00338-006-0112-y)

7. Van Dam, A. A., Beveridge, M. C. M., Azim, M. E. & Verdegem, M. C. J. 2002 The potential of fish production based on periphyton. *Rev. Fish Biol. Fish.* **12**, 1–31. (doi:10.1023/A:1022639805031)

8. Goatley, C. H. R. & Bellwood, D. R. 2010 Biologically mediated sediment fluxes on coral reefs: Sediment removal and off-reef transportation by the surgeonfish Ctenochaetus striatus. *Mar. Ecol. Prog. Ser.* **415**, 237–245. (doi:10.3354/meps08761)

9. Marshell, A. & Mumby, P. J. 2012 Revisiting the functional roles of the surgeonfish *Acanthurus nigrofuscus* and *Ctenochaetus striatus*. *Coral Reefs* **31**, 1093–1101. (doi:10.1007/s00338-012-0931-y)

10. Choat, J. H., Robbins, W. D. & Clements, K. D. 2004 The trophic status of herbivorous fishes on coral reefs: II. Food processing modes and trophodynamics. *Mar. Biol.* **145**, 445–454. (doi:10.1007/s00227-004-1341-7)

11. Hatcher, B. & Larkum, A. 1983 An experimental analysis of factors controlling the standing crop of the epilithic algal community on a coral reef. *J. Exp. Mar. Bio. Ecol.* **69**, 61–84.

12. Hay, M. E., Colbum, T. & Downing, D. 1983 Spatial and temporal patterns in herbivory on a Caribbean fringing reef: the effects on plant distribution. *Oecologia* **58**, 299–308.

13. Bellwood, D. R. & Choat, J. H. 1990 A functional analysis of grazing in parrotfishes (family Scaridae): the ecological implications. *Environ. Biol. Fishes* **28**, 189–214. (doi:10.1007/BF00751035)

14. Steneck, R. S. 1988 Herbivory on coral reefs: A synthesis. In *Proceedings of the 6th International Coral Reef Symposium*, pp. 37–49.

15. McCauley, D. J., Young, H. S., Guevara, R., Williams, G. J., Power, E. A., Dunbar, R. B., Bird, D. W., Durham, W. H. & Micheli, F. 2014 Positive and negative effects of a threatened parrotfish on reef ecosystems. *Conserv. Biol.* **28**, 1312–1321. (doi:10.1111/cobi.12314)

16. Gove, J. M., Williams, G. J., McManus, M. A., Heron, S. F., Sandin, S. A., Vetter, O. J. & Foley, D. G. 2013 Quantifying climatological ranges and anomalies for Pacific coral reef ecosystems. *PLoS One* **8**, e61974. (doi:10.1371/journal.pone.0061974)

17. Graham, N. A. J., Dulvy, N. K., Jennings, S. & Polunin, N. V. C. 2005 Size-spectra as indicators of the effects of fishing on coral reef fish assemblages. *Coral Reefs* **24**, 118–124. (doi:10.1007/s00338-004-0466-y)

18. Dulvy, N. K., Polunin, N. V., Mill, a C. & Graham, N. A. 2004 Size structural change in lightly exploited coral reef fish communities: evidence for weak indirect effects. *Can. J. Fish. Aquat. Sci.* **61**, 466–475. (doi:10.1139/f03-169)

19. Williams, I. D., Walsh, W. J., Schroeder, R. E., Friedlander, A. M., Richards, B. L. & Stamoulis, K. A. 2008 Assessing the importance of fishing impacts on Hawaiian coral reef fish assemblages along regional-scale human population gradients. *Environ. Conserv.* **35**, 261. (doi:10.1017/S0376892908004876)

20. D'agata, S. et al. 2014 Human-mediated loss of phylogenetic and functional diversity in coral reef fishes. *Curr. Biol.* **24**, 555–60. (doi:10.1016/j.cub.2014.01.049)

21. Williams, I. D., Baum, J. K., Heenan, A., Hanson, K. M., Nadon, M. O. & Brainard, R. E. 2015 Human, oceanographic and habitat drivers of central and western Pacific coral reef fish assemblages. *PLoS One* **10**, e0120516. (doi:10.1371/journal.pone.0120516)

22. Brewer, T. D., Cinner, J. E., Green, A. & Pressey, R. L. 2013 Effects of human population density and proximity to markets on coral reef fishes vulnerable to extinction by fishing. *Conserv. Biol.* **27**, 443–452. (doi:10.1111/j.1523-1739.2012.01963.x)

23. Cinner, J. E., Graham, N. A. J., Huchery, C. & Macneil, M. A. 2013 Global effects of local human population density and distance to markets on the condition of coral reef fisheries. *Conserv. Biol.* **27**, 453–8. (doi:10.1111/j.1523-1739.2012.01933.x)

24. Maire, E. et al. 2016 How accessible are coral reefs to people? A global assessment based on travel time. *Ecol. Lett.* **19**, 351–360. (doi:10.1111/ele.12577)

25. Brewer, T. D., Cinner, J. E., Fisher, R., Green, A. & Wilson, S. K. 2012 Market access, population density, and socioeconomic development explain diversity and functional group biomass of coral reef fish assemblages. *Glob. Environ. Chang.* **22**, 399–406. (doi:10.1016/j.gloenvcha.2012.01.006)

26. Pinca, S., Kronen, M., Magron, F., McArdle, B., Vigliola, L., Kulbicki, M. & Andréfouët, S. 2012 Relative importance of habitat and fishing in influencing reef fish communities across seventeen Pacific Island Countries and Territories. *Fish Fish.* **13**, 361–379. (doi:10.1111/j.1467-2979.2011.00425.x)

27. Kittinger, J. N. et al. 2015 From reef to table: social and ecological factors affecting coral reef fisheries, artisanal seafood supply chains, and seafood security. *PLoS One* **10**, e0123856. (doi:10.1371/journal.pone.0123856)

28. Cuetos-Bueno, J. & Houk, P. 2014 Re-estimation and synthesis of coral-reef fishery landings in the Commonwealth of the Northern Mariana Islands since the 1950s suggests the decline of a common resource. *Rev. Fish Biol. Fish.* , 179–194. (doi:10.1007/s11160-014-9358-6)

29. Williams, I. D., Hongguang, M. & Williams, Ivor; Ma, H. 2013 Estimating catch weight of reef fish species using estimation and intercept data from the Hawaii marine recreational fishing survey. Pacific Islands Fish. Sci. Cent. Admin. Rep. H-13-04, 53 p.

30. Mora, C. et al. 2011 Global human footprint on the linkage between biodiversity and ecosystem functioning in reef fishes. *PLoS Biol.* **9**, e1000606. (doi:10.1371/journal.pbio.1000606)

31. Cinner, J. E. et al. 2016 Bright spots among the world's coral reefs. *Nature* (doi:10.1038/nature18607)

32. MacNeil, M. A. et al. 2015 Recovery potential of the world's coral reef fishes. *Nature* (doi:10.1038/nature14358)

33. Zuur, A. F. 2012 *Beginner's guide to generalized additive models with R*. Newburgh: Highland Statistics Ltd.

34. Bartoń, K. 2015 MuMIn: Multi-model inference. Available at: http://cran.r-project.org/package=MuMin.

35. Dulvy, N. K. & Polunin, N. V. C. 2004 Using informal knowledge to infer human-induced rarity of a conspicuous reef fish. *Anim. Conserv.* **7**, 365–374. (doi:10.1017/S1367943004001519)

36. Bellwood, D. R., Hoey, A. S. & Choat, J. H. 2003 Limited functional redundancy in high diversity systems: Resilience and ecosystem function on coral reefs. *Ecol. Lett.* **6**, 281–285. (doi:10.1046/j.1461-0248.2003.00432.x)

37. Hoey, A. S. & Bellwood, D. R. 2008 Cross-shelf variation in the role of parrotfishes on the Great Barrier Reef. *Coral Reefs* **27**, 37–47. (doi:10.1007/s00338-007-0287-x)

38. Zgliczynski, B. J., Williams, I. D., Schroeder, R. E., Nadon, M. O., Richards, B. L. & Sandin, S. a. 2013 The IUCN Red List of Threatened Species: An assessment of coral reef fishes in the US Pacific Islands. *Coral Reefs* **32**, 637–650. (doi:10.1007/s00338-013-1018-0)

39. Heenan, A. et al. 2016 Ecosystem monitoring for ecosystem-based management: Using a polycentric approach to balance information trade-offs. *J. Appl. Ecol.* (doi:10.1111/1365-2664.12633)

40. Thompson, A. A. & Mapstone, B. D. 2002 Intra- versus inter-annual variation in counts of reef fishes and interpretations of long-term monitoring studies. *Mar. Ecol. Prog. Ser.* **232**, 247–257. (doi:10.3354/meps232247)

41. Donaldson, T. J. & Dulvy, N. K. 2004 Threatened fishes of the world : *Bolbometopon muricatum* (Valenciennes 1840) (Scaridae). *Environ. Biol. Fishes* **70**, 373.

42. Bellwood, D. R., Hoey, A. S. & Hughes, T. P. 2012 Human activity selectively impacts the ecosystem roles of parrotfishes on coral reefs. *Proc. R. Soc. B* **279**, 1621–1629. (doi:10.1098/rspb.2011.1906)

43. Bates, D., Mächler, M., Bolker, B. M. & Walker, S. C. 2015 Fitting linear mixed-effects models using lme4. *J. Stat. Softw.* **67**, 1–48. (doi:10.18637/jss.v067.i01)
